# Orosomucoid 1 Attenuates Doxorubicin-Induced Oxidative Stress and Apoptosis in Cardiomyocytes via Nrf2 Signaling

**DOI:** 10.1155/2020/5923572

**Published:** 2020-10-19

**Authors:** Xiaoli Cheng, Dan Liu, Ruinan Xing, Haixu Song, Xiaoxiang Tian, Chenghui Yan, Yaling Han

**Affiliations:** ^1^Department of Cardiology, Shengjing Hospital of China Medical University, Shenyang, Liaoning Province 110004, China; ^2^Department of Cardiology and Cardiovascular Research Institute of PLA, General Hospital of Northern Theater Command, Shenyang, Liaoning Province 110016, China

## Abstract

Doxorubicin (DOX) is an effective anticancer drug, but its therapeutic use is limited by its cardiotoxicity. The principal mechanisms of DOX-induced cardiotoxicity are oxidative stress and apoptosis in cardiomyocytes. Orosomucoid 1 (ORM1), an acute-phase protein, plays important roles in inflammation and ischemic stroke; however, the roles and mechanisms of ORM1 in DOX-induced cardiotoxicity remain unknown. Therefore, in the present study, we aimed to investigate the function of ORM1 in cardiomyocytes experiencing DOX-induced oxidative stress and apoptosis. A DOX-induced cardiotoxicity animal model was established in C57BL/6 mice by administering an intraperitoneal injection of DOX (20 mg/kg), and the control group was intraperitoneally injected with the same volume of sterilized saline. The effects were assessed after 7 d. Additionally, H9c2 cells were stimulated with DOX (10 *μ*M) for 24 h. The results showed decreased ORM1 and increased oxidative stress and apoptosis after DOX stimulation in vivo and in vitro. ORM1 overexpression significantly reduced DOX-induced oxidative stress and apoptosis in H9c2 cells. ORM1 significantly increased the expression of nuclear factor-like 2 (Nrf2) and its downstream protein heme oxygenase 1 (HO-1) and reduced the expression of the lipid peroxidation end product 4-hydroxynonenal (4-HNE) and the level of cleaved caspase-3. In addition, Nrf2 silencing reversed the effects of ORM1 on DOX-induced oxidative stress and apoptosis in cardiomyocytes. In conclusion, ORM1 inhibited DOX-induced oxidative stress and apoptosis in cardiomyocytes by regulating the Nrf2/HO-1 pathway, which might provide a new treatment strategy for DOX-induced cardiotoxicity.

## 1. Introduction

Heart failure is a prevalent disease worldwide, representing a severe manifestation and end stage of most heart diseases. The number of patients with heart failure in China has reached 1-2% of the total population. Heart failure not only significantly decreases the quality of life of patients and causes a substantial economic burden on patient families and society but is also associated with a high mortality rate. Over 50% of patients with chronic heart failure die within five years of diagnosis. Therefore, heart failure is also known as “the final battlefield of cardiovascular disease of the 21st century”.

The incidence rate of cancer is more than one-third worldwide, and cardiovascular disease is the two causes of death in developed countries. Doxorubicin (DOX), a cardiotoxic anthracycline chemotherapy drug, was first isolated from *Streptomyces peucetius caesius* in 1967 [1, 2]. DOX plays an important role in the treatment of many cancers, as 32% of patients with breast cancer, 57-70% of elderly patients with lymphoma, and 50-60% of children with cancer have been treated with anthracycline drugs [3–6]. DOX has been included in the World Health Organization (WHO) model list of essential medicines, as a milestone of cancer therapy development and one of the most commonly used antitumor anthracycline antibiotics.

However, the cardiotoxic effects of DOX limit its use in cancer patients with irreversible degenerative cardiomyopathy and heart failure. More than half of the elderly patients and children who survive lymphoma and other cancers, respectively, show a high risk of cardiotoxicity after DOX treatment [7]. Therefore, it is essential to study DOX-induced myocardial injury to increase survival rates and improve the quality of life of patients. Numerous studies have shown that oxidative stress and apoptosis are important mechanisms of DOX-induced myocardial injury [8, 9]. Therefore, an effective antioxidant and antiapoptotic agent that improves DOX-induced myocardial injury is urgently needed.

Orosomucoid 1 (ORM1) is an acute-phase protein that was first discovered by Tokita and Schmid more than 100 years ago. It is mainly synthesized in the liver, but many extrahepatic tissues have also been reported to produce ORM1 under various conditions [10, 11]. ORM1 performs various activities, acting as an acute-phase reactant and disease marker, regulating immunity, maintaining the capillary barrier function, regulating sphingomyelin metabolism, and scavenging reactive oxygen species (ROS) [12–14]. However, the current understanding of ORM1 is limited [15, 16], and its role in cardiovascular disease is not clear.

Nuclear factor-like 2 (Nrf2) is an important member of the cap collar family of basic leucine zipper transcription factors; it plays a role in the antioxidant defense system by regulating the expression of antioxidant enzymes [17, 18]. During conditions of oxidative stress, Nrf2 is activated, translocated to the nuclear region, and combined with the antioxidant response element located in the promoter region of phase II antioxidant enzyme genes, such as heme oxygenase 1 (*HO-1*). It results in the detoxification of 4-hydroxylnonenal (4-HNE) and a decrease in ROS level, which releases the level of apoptosis [19, 20]. In previous studies, it was found that DOX decreases the expression of Nrf2, resulting in an increase in oxidative stress and cell apoptosis [21]. However, it is not clear whether ORM1 plays a protective role during oxidative stress and cell apoptosis by regulating Nrf2.

In our study, we aimed to investigate the potential relationship between ORM1 and Nrf2 and clarify the roles of ORM1 in the oxidative stress and cell apoptosis resulting from DOX-induced cardiomyocyte toxicity.

## 2. Materials and Methods

### 2.1. Animals and In Vivo Experimental Design

Eight-week-old male C57BL/6J mice were acquired from Southern Animal Model Co., Ltd. (Nanjing, China) and housed in a nonpathogenic animal facility at an ambient temperature of 23 ± 2°C and on a 12 h dark/light cycle for 2 wk. DOX (Sigma, USA) was dissolved in sterilized saline to a final concentration of <2%. Forty mice were randomly divided into the following two groups with 20 mice in each group: control (saline) and DOX (20 mg/kg DOX). Mice in the DOX group were intraperitoneally injected with 10 mg/kg DOX on the first and fourth days, leading to a cumulative DOX dose of 20 mg/kg. Mice in the control group were intraperitoneally injected with the same volume of saline at the same times. These doses of DOX and sterilized saline were selected based on previous studies [22]. Seven days after the first DOX injection, all mice were euthanized. All animal experiments complied with the “Guiding Principles for the Care of Experimental Animals” and “Guidelines for the Care and Use of Experimental Animals” (NIH publication 86-23, revised 1985). Animal care and procedures were approved by the Committee on the Care and Use of Laboratory Animals of the General Hospital of Northern Theater Command.

### 2.2. Cell Culture, Transfection, and In Vitro Experimental Design

The H9c2 cell line was purchased from the Chinese Academy of Sciences, Shanghai Institute for the Cell Resource Center and cultured in 5% CO_2_ at 37°C. Adenoviral vectors (Ad-control and Ad-ORM1) were purchased from Hanbio Biotechnology (China). Nrf2 siRNA and control siRNA were purchased from Thermo Scientific (USA). H9c2 cells were seeded onto 6-well plates (2 × 10^5^ cells per well), and fetal bovine serum (FBS, USA) was added to 2 mL of normal culture medium without antibiotics. After the cells reached a confluence of 60-70%, they were transfected using Lipo iMAX (Thermo Scientific, USA) to control siRNA or target siRNA double strands (100 pM). After 48 h of transfection, the cells were collected for further experiments. DOX was dissolved in sterile saline.

Before the experiment, sterile saline was used as a control. In the preliminary in vitro experiment, the cells were divided into the following four groups: (1) control, (2) ORM1 (100 MOI) treated (ORM1), (3) DOX (10 *μ*M) treated (DOX), and (4) DOX+ORM1 treated (DOX+ORM1). In the subsequent in vitro experiments, the cells were divided into the following groups: (1) control, (2) Nrf2 siRNA treated (Nrf2), (3) DOX treated (DOX), (4) DOX+Nrf2 siRNA treated, (5) DOX+ORM1 treated, and (6) DOX+ORM1+Nrf2 siRNA treated. After treatment, the cells were collected for further analyses.

### 2.3. Echocardiography

Seven days after the first DOX injection, the mice were anesthetized with 1.5% isoflurane to stabilize the heart rate at 400-500 bpm. The size and function of the hearts were measured by M-mode echocardiography using the ms-400 linear transducer echocardiography system (Visual Sonics Vevo 2100, Toronto, CA). All measurements are the average of five consecutive heartbeat cycles.

### 2.4. Measurement of Plasma Levels of Lactate Dehydrogenase (LDH) and Creatine Kinase Isoenzyme (CKMB)

Blood samples were taken from mice in the control and DOX groups and poured into EDTA tubes to prevent clotting. Plasma was separated by centrifugation. LDH and CKMB levels in plasma were determined using the standard enzyme-linked immunosorbent assay (ELISA) kit (Roche, Switzerland) and presented in U/l. All procedures were performed according to the manufacturer's instructions.

### 2.5. Wheat Germ Agglutinin (WGA) Staining

Hearts were collected from five mice in the control and DOX groups, respectively, and then fresh-frozen heart tissues were stained with WGA (Sigma, USA) according to the manufacturer's instructions (200 cross-sectional areas per mouse). Fluorescence images were examined using a Zeiss Axio Imager 2 microscope (Zeiss, Germany).

### 2.6. Terminal Deoxynucleotidyl Transferase dUTP Nick End Labeling (TUNEL) Assay

Hearts were collected from five mice in the control and DOX groups, respectively. Frozen heart tissues were cut into 5 *μ*m thick sections, and H9c2 cells were cultured on cover glass. H9c2 cells were transfected with ORM1 virus, Nrf2 siRNA, or the corresponding control. After 48 h of transfection, cells were stimulated with DOX for 24 h and fixed with 4% paraformaldehyde. Apoptosis in myocardial tissues was analyzed using a TUNEL staining kit (Roche, Switzerland), and H9c2 cells were analyzed using a different TUNEL staining kit (Abcam, UK) according to the manufacturer's instructions. Fluorescence images were examined using a Zeiss Axio Imager 2 microscope (Zeiss, Germany).

### 2.7. Liquid Chromatography-Tandem Mass Spectrometry (LC/MS)

The hearts were collected from mice in the control and DOX groups, and three mice from each group were sent to Bio Miao Biological and processed according to the corresponding procedure. The peptide mixtures in each group of samples were labeled with different isobaric tags for relative and absolute quantification (iTRAQ) reagents; equal amounts of the labeled peptides in each sample were prepared. Finally, LC/MS detection and analysis were performed.

### 2.8. Immunohistochemical Staining

Mouse hearts were fixed in 4% paraformaldehyde (pH 7.4) overnight, embedded in paraffin, and continuously sectioned at a thickness of 5 *μ*m. For dewaxing, sections were sealed with phosphate-buffered saline (PBS) containing 5% normal target serum and 1% bovine serum albumin (BSA) and then incubated overnight at 4°C under humidified conditions with anti-ORM1 (Abcam, UK), anti-Nrf2 (Abcam, UK), anti-HO-1 (Abcam, UK), anti-4-HNE (Abcam, UK), and anticleaved caspase-3 (Cell Signaling Technology, USA). A routine histological examination was performed using light microscopy. Images were examined with a Zeiss Axio Imager 2 microscope (Zeiss, Germany).

### 2.9. Analysis of Cell Viability

Cell survival rate was determined using the Cell Counting Kit-8 (CCK-8, Beyotime, China) according to the manufacturer's instructions. H9c2 cells (1 × 10^4^) were seeded onto 96-well plates and subjected to the treatments described above. CCK-8 solution (10 *μ*L) was added to 100 *μ*L of culture medium in each well and incubated for 2 h at 37°C, before the optical density (OD) was measured at 450 nm with a Thermo Multiskan FC microplate reader (Thermo, USA). Cell survival rates were expressed as the ratio of the OD value of the experimental well to that of the control well. Each treatment was performed six times. Cell survival rates were expressed as the ratio of the OD value of the experimental well to that of the control well.

### 2.10. Malondialdehyde (MDA) Determination

To analyze oxidative stress parameters, we examined the MDA content. H9c2 cells (1 × 10^4^) were seeded onto 96-well plates and subjected to the treatments described above. Blood samples were collected from mice in the control and DOX groups. We determined the MDA content using a commercially available MDA detection kit (Beyotime, China) according to the manufacturer's instructions.

### 2.11. Dichlorodihydrofluorescein Diacetate (DCFH-DA) Assay

The production of ROS was established using a DCFH-DA staining kit (Beyotime, China). H9c2 cells were seeded onto 96-well plates and subjected to the treatments described above, then incubated with 10 *μ*M DCFH-DA for 30 min in the dark at 37°C. Fluorescence intensities were immediately measured by spectrophotometry using a microplate reader; the excitation and emission wavelengths were 488 and 522 nm, respectively. The fluorescence intensity of the control group was set to 100%. The accumulation of ROS in H9c2 cells was analyzed by confocal microscopy (red staining), and the fluorescence intensity of DCFH-DA was quantified using the Image Pro Plus software (Media Cybernetics Inc., USA).

### 2.12. Polymerase Chain Reaction (PCR) Analysis

Trizol (Invitrogen, USA) was used to extract total RNA from heart tissues and cells, and the concentration and purity of RNA were determined by spectrophotometry. The PrimeScript RT Kit (Takara, Japan) was used to synthesize cDNA according to the manufacturer's instructions. Quantitative PCR was performed using the CFX96 Real-Time System (Bio-Rad) to detect differences in gene expression. Relative gene expression of each protein of interest was calculated using the 2^-∆∆CT^ method and normalized to GAPDH expression. All reactions were performed in triplicate, and the specificity was monitored by melting curve analysis. The primers were purchased from RiboBio (China) (see S-Fig4 for the PCR primers used).

### 2.13. Western Blot Analysis

After collecting cells and heart samples from the different treatment groups and extracting proteins, the protein concentrations were determined using a bicinchoninic acid (BCA) protein assay (Thermo Scientific, USA). The samples from each group were separated by sodium dodecyl sulfate polyacrylamide gel electrophoresis (SDS-PAGE) and transferred to a polyvinylidene fluoride (PVDF) membrane. The membrane was blocked by incubation with 5% skimmed milk (containing 0.1% Tween-20) for 2 h at room temperature. After incubation, the membrane was further incubated with the primary antibodies anti-ORM1 (Abcam, UK), anti-Nrf2 (Abcam, UK), anti-HO-1 (Abcam, UK), anti-4-HNE (Abcam, UK), and anticleaved caspase-3 (Cell Signaling Technology, USA). Next, the membrane was washed in Tris-buffered saline Tween (TBST) and incubated with the corresponding secondary antibody (Abcam; at a dilution of 1 : 5000) for 2 h at room temperature. The Amersham Imager 680 (GE, USA) was used to detect the bands, and the signal was quantified.

### 2.14. Statistical Analysis

The data are expressed as the mean ± standard error of the mean (SEM). Data were analyzed using the SPSS 19.0 statistical software (SPSS Inc., USA). Differences between two groups were calculated using the *t*-test, and one-way analysis of variance (ANOVA) with Tukey's post hoc test was used when comparing multiple groups. Differences with a *P* value < 0.05 were considered significant.

## 3. Results

### 3.1. DOX Caused Cardiac Dysfunction and Heart Injury in C57BL/6 Mice

**Figure 1:**
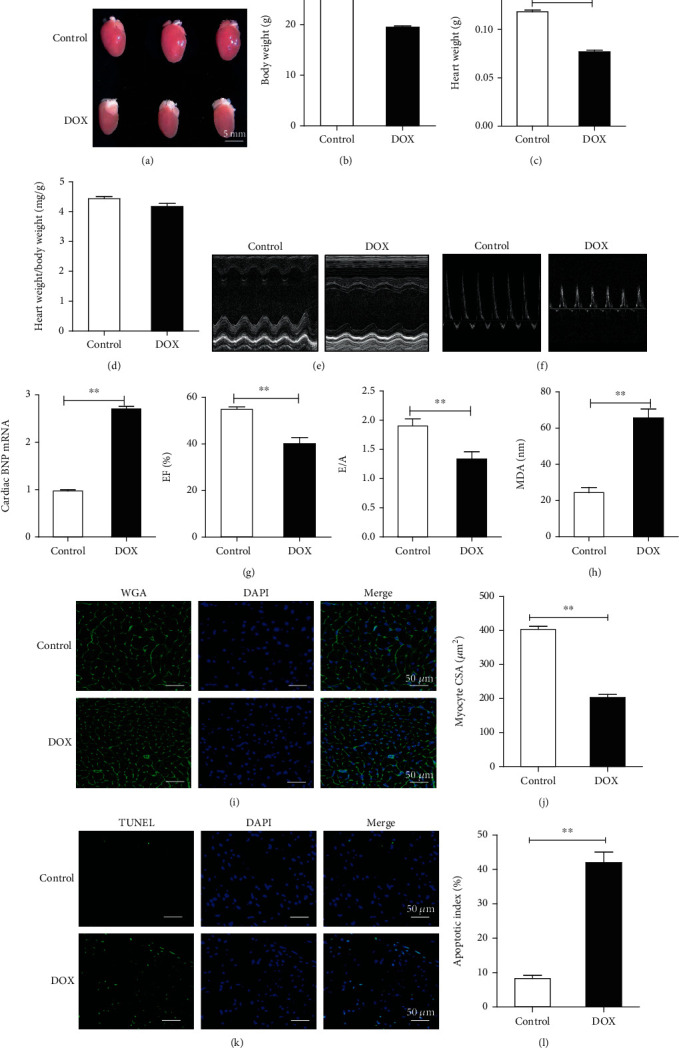
Effects of doxorubicin (DOX) on C57BL/6 mice. (a) General pictures of the heart. (b) Body weight. (c) Heart weight. (d) Heart weight/body weight. (e, f) Systolic and diastolic functions in both groups (*n* = 20). (g) B-type natriuretic peptide (BNP) levels in mice detected using polymerase chain reaction (PCR) (*n* = 10). (h) Malondialdehyde (MDA) levels in both groups (*n* = 10). (i, j) Myocardial cell size evaluated using wheat germ agglutinin (WGA) staining (*n* = 5). (k, l) Terminal deoxynucleotidyl transferase dUTP nick end labeling (TUNEL) staining (*n* = 5). Data are expressed as the mean ± standard error of the mean (SEM). ^∗∗^*P* < 0.01.

All experimental mice were weighed to determine weight loss during each treatment. The results showed that the weight of DOX-treated mice was higher than that of the control group. After sacrificing, it was found that the hearts of DOX-treated mice were significantly atrophic, and the heart weight was significantly reduced compared to that of the normal group, indicating DOX-induced cardiac atrophy (Figures 1(a)–1(d)). DOX caused acute cardiac dysfunction in male mice, leading to a significant decrease in systolic and diastolic functions relative to those of the control group (Figures 1(e) and 1(f), Supplementary Figure 1a). To determine the levels of myocardial injury caused by DOX, we tested the serum LDH and CKMB levels and found that the difference was statistically significant between the DOX-treated and control groups (Supplementary Figure 1b-c). To evaluate cell damage induced by DOX, we measured the B-type natriuretic peptide (BNP) and A-type natriuretic peptide(ANP) levels 7 d after DOX administration and found that the mRNA levels of BNP and ANP were significantly increased in DOX-treated mice, indicating obvious heart failure (Figure 1(g), Supplementary Figure 1d).

According to previous studies, one of the main mechanisms of DOX-mediated myocardial injury is oxidative stress injury. Therefore, we examined the serum MDA levels in the mice and found that the level in the DOX group was significantly higher than that in the control group (Figure 1(h)). To evaluate the DOX-induced damage to cardiomyocytes, we stained murine heart sections with WGA and found that cardiomyocytes from DOX-treated mice were significantly reduced, which is in accordance with the observed atrophy (Figures 1(i) and 1(j)). To evaluate the level of cardiomyocyte apoptosis, we conducted TUNEL staining of the myocardial tissues of the mice. The results showed that compared with the control group, the DOX group exhibited a significantly higher apoptosis index (Figures 1(k) and 1(l)).

### 3.2. DOX Treatment Evoked the Downregulation of ORM1 and Upregulation of Oxidative Stress and Apoptosis in Murine Myocardial Tissue

**Figure 2:**
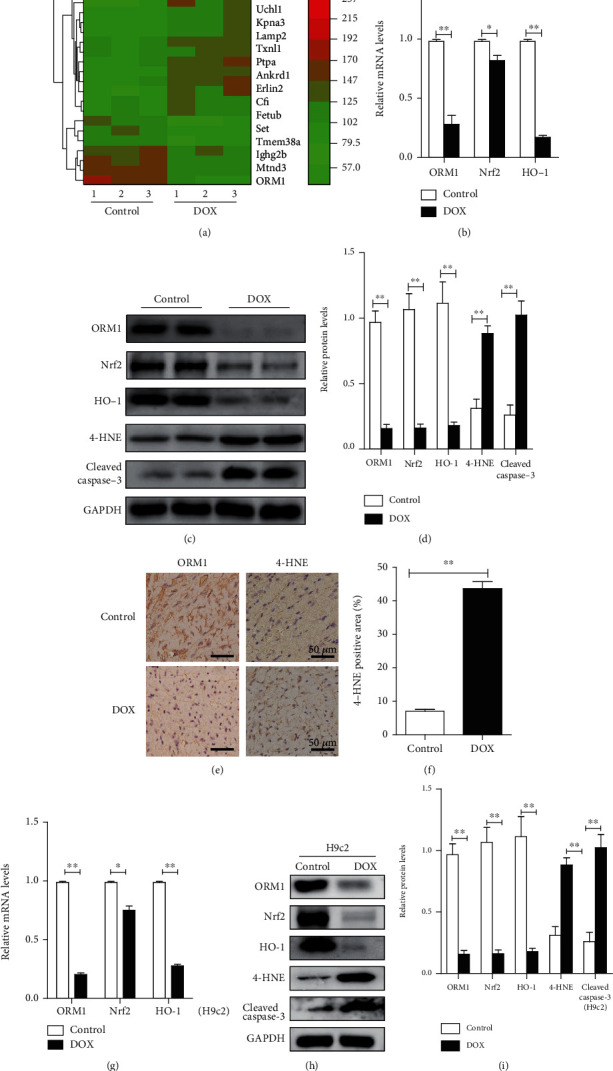
Downregulation of ORM1 and upregulation of oxidative stress and apoptosis in the DOX-induced cardiomyopathy model in vitro and in vivo. (a) Differentially expressed candidate proteins in control and DOX-treated mice analyzed using LC/MS technology. Low expression is depicted in green, and high expression is depicted in red. (b) mRNA levels of ORM1, Nrf2, and HO-1 in the hearts of mice in the DOX-treated groups (*n* = 6). (c, d) Western blot analysis of ORM1, Nrf2, HO-1, 4-HNE, and cleaved caspase-3 (*n* = 6). (e, f) Immunohistochemical staining images of ORM1- and 4-HNE-stained heart sections from control and DOX-treated mice (*n* = 6). (g) mRNA levels of ORM1, Nrf2, and HO-1 in DOX-treated H9c2 cells (10 *μ*M and 24 h) and the control group (*n* = 3). (h, i) Western blot analysis of ORM1, Nrf2, HO-1, 4-HNE, and cleaved caspase-3 in DOX-treated H9c2 cells (10 *μ*M and 24 h) and the control group (*n* = 3). Data are expressed as the mean ±standard error of the mean (SEM). ^∗∗^*P* < 0.01 and ^∗^*P* < 0.05.

First, we collected the hearts from control and DOX-treated mice and performed LC/MS analysis. The heat map shows differentially expressed genes between the cardiac tissues of control and DOX-treated mice. Low expression is depicted in green, and high expression is depicted in red. We found that ORM1 levels were significantly decreased in DOX-treated mice (Figure 2(a)). We then examined the mRNA levels of ORM1, Nrf2, and HO-1 in vivo. Compared with those in the control group, the mRNA levels of ORM1, Nrf2, and HO-1 were significantly reduced in the DOX group (Figure 2(b)). The protein levels of ORM1, Nrf2, and HO-1 were also significantly reduced in the DOX group, which was consistent with the effect on the mRNA levels (Figures 2(c) and 2(d)). In addition, 4-HNE is the downstream molecule of HO-1, which can cause extensive oxidative damage and cell apoptosis, and cleaved caspase-3 is a marker of cell apoptosis. Therefore, we chose to examine 4-HNE and cleaved caspase-3 to evaluate the effects of DOX on the level of oxidative stress and cell apoptosis. Compared with those in the control group, the protein levels of 4-HNE and cleaved caspase-3 were significantly increased in the DOX group (Figures 2(c) and 2(d)). The results of immunohistochemical staining of the myocardia from DOX-treated and control mice confirmed the above findings (Figures 2(e) and 2(f)).

### 3.3. DOX Treatment Produced the Downregulation of ORM1 and Upregulation of Oxidative Stress and Apoptosis in H9c2 Cells

In H9c2 cells, different concentrations of DOX (0, 2, 5, and 10 *μ*M) were used to examine the effect of DOX on cell viability. The results showed that DOX significantly reduced cell viability in a concentration-dependent manner; the effect was most obvious when the concentration was 10 *μ*M (Supplementary Figure 2). Therefore, 10 *μ*M DOX was used in subsequent experiments.

The mRNA and protein levels of ORM1, Nrf2, and HO-1 in H9c2 cells treated with DOX were significantly decreased (Figures 2(g)–2(i)). In addition, the levels of 4-HNE and cleaved caspase-3 were significantly increased in H9c2 cells after DOX stimulation (Figures 2(h) and 2(i)). These results were consistent with the in vivo results and indicated that decreased levels of ORM1 might play an important role in DOX-induced cardiotoxicity.

### 3.4. ORM1 Reduced DOX-Induced Oxidative Stress and Apoptosis in H9c2 Cells

To explore the protective effect of ORM1 against DOX in H9c2 cells in vitro, the effect of ORM1 on the viability of H9c2 cells was measured. A concentration of ORM1 below 100 MOI produced no cytotoxic effects. However, a higher concentration slightly reduced the viability of H9c2 cells (Supplementary Figure 3a). Cotreatment of 10 *μ*M DOX and either 10, 30, 50, or 100 MOI ORM1 caused a concentration-dependent increase in cell viability compared to that of the control group (Supplementary Figure 3b). Further experiments were carried out using 10 *μ*M DOX.

**Figure 3:**
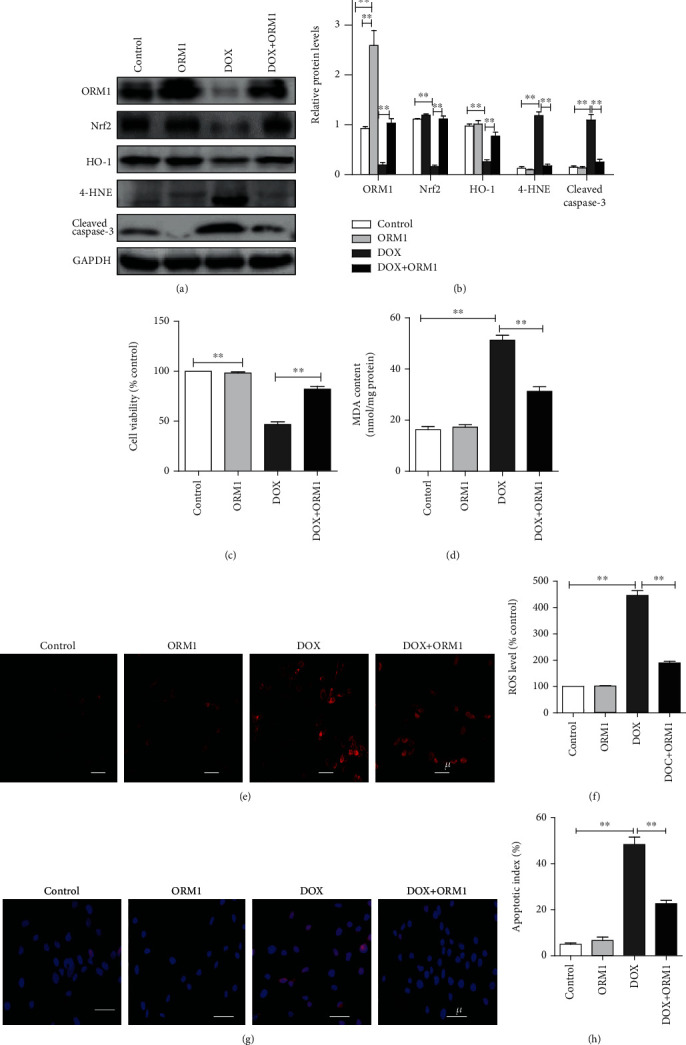
ORM1 reduces doxorubicin- (DOX-) induced oxidative stress and apoptosis in H9c2 cells. (a, b) Western blot analysis of ORM1, Nrf2, HO-1, 4-HNE, and cleaved caspase-3. (c) Cell survival rate analysis using the Cell Counting Kit 8 (CCK-8). Cell survival rate is expressed as the optical density (OD) value (% control). (d) Cellular malondialdehyde (MDA) content. (e, f) Fluorescence image (red fluorescence) of reactive oxygen species (ROS) measured using dichlorodihydrofluorescein diacetate (DCFH-DA). (g, h) Terminal deoxynucleotidyl transferase dUTP nick end labeling (TUNEL) staining images with calculated apoptosis indices. Data are expressed as the mean ± standard error of the mean (SEM); *n* = 6. ^∗∗^*P* < 0.01.

Next, we more closely examined the effects of 100 MOI ORM1 on H9c2 cells treated with DOX. Western blot analysis demonstrated that the addition of exogenous ORM1 reduced the oxidative level and apoptosis index in cardiomyocytes, reversing the DOX-induced effects (Figures 3(a) and 3(b)). The CCK-8 assay was utilized to further examine the protective effects of ORM1 in H9c2 cells. The results showed that the cell survival rate significantly increased in DOX+ORM1-treated cells compared with that in cells treated with DOX only (Figure 3(c)).

MDA is a parameter of oxidative stress, and its content in cells was significantly upregulated by DOX (Figure 3(d)). Under various pathological conditions, the initiation of oxidative stress and the overproduction of ROS play a key role in the development of cardiac dysfunction. A DCFH-DA assay was utilized to analyze the production of cellular ROS, and the fluorescence intensity increased with the production of reactive metabolites. The combination of ORM1 and DOX effectively inhibited ROS production (Figures 3(e) and 3(f)). A subsequent TUNEL assay of H9c2 cells showed that DOX significantly increased apoptosis, while ORM1 produced the opposite effect (Figures 3(g) and 3(h)).

### 3.5. Nrf2 Knockdown Reversed the Protective Effects of ORM1 in DOX-Treated H9c2 Cells

**Figure 4:**
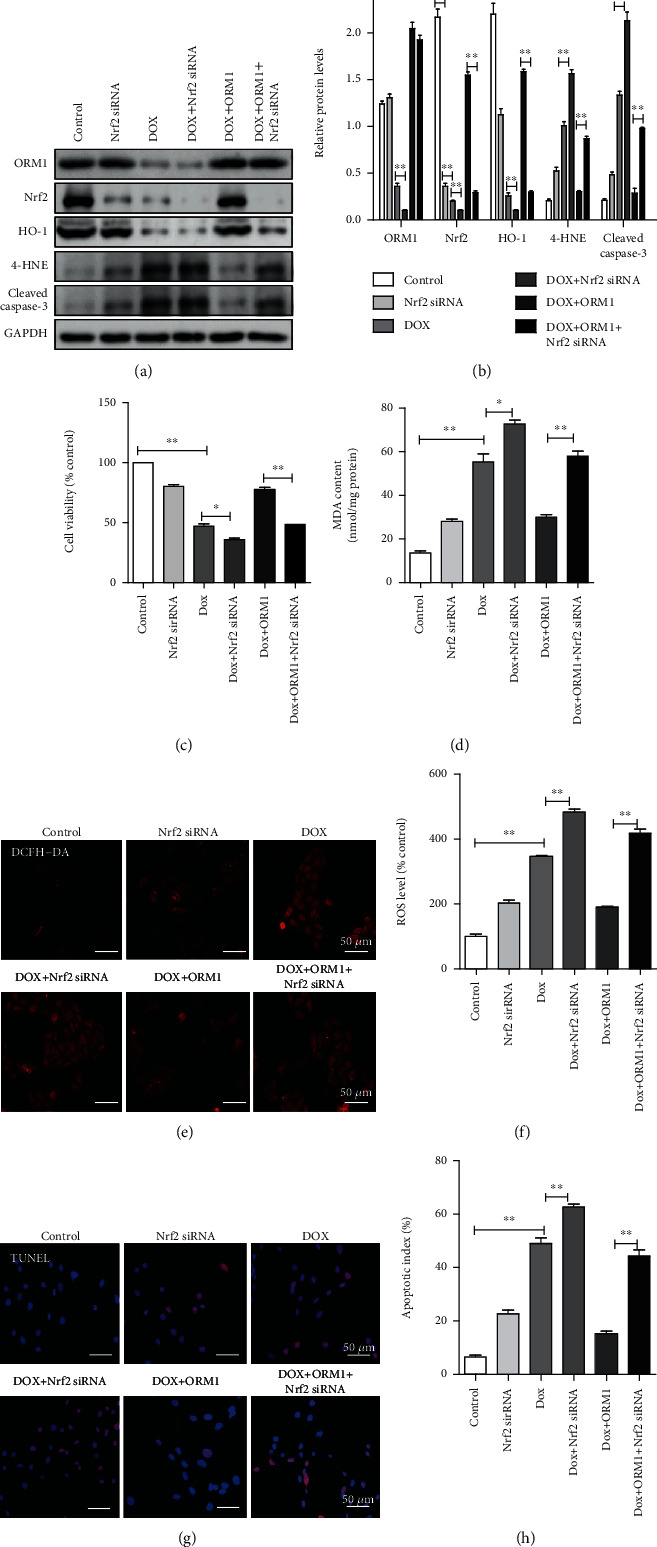
Nrf2 knockdown reverses the protective effects of ORM1 in doxorubicin- (DOX-) treated H9c2 cells. (a, b) Western blot analysis of ORM1, Nrf2, HO-1, 4-HNE, and cleaved caspase-3. (c) Cell survival analysis using the Cell Counting Kit 8 (CCK-8). (d) Cellular malondialdehyde (MDA) content. (e, f) Fluorescence image (red fluorescence) of reactive oxygen species (ROS) measured using dichlorodihydrofluorescein diacetate (DCFH-DA). (g, h) Terminal deoxynucleotidyl transferase dUTP nick end labeling (TUNEL) staining images with calculated apoptosis indices. Data are expressed as the mean ± standard error of the mean (SEM); *n* = 6. ^∗∗^*P* < 0.01 and ^∗^*P* < 0.05.

To study the role of the Nrf2-mediated transcription network in the DOX-induced cardioprotective effects of ORM1, Nrf2 was knocked down by specific Nrf2 siRNA. Western blot analysis showed that Nrf2 knockdown did not affect the ORM1 level, it downregulated HO-1 expression and upregulated 4-HNE and cleaved caspase-3 levels in the DOX+ORM1+Nrf2 siRNA group compared to the corresponding levels in the DOX+ORM1 group (Figures 4(a) and 4(b)). CCK-8 analysis showed that compared with cells expressing Nrf2, Nrf2-knockdown cells exhibited an aggravated reduction of cell viability in DOX group, while silencing Nrf2 significantly reduced cell viability in the DOX+ORM1+Nrf2 siRNA group compared to the corresponding levels in the DOX+ORM1 group in H9c2 cells (Figure 4(c)). Similarly, silencing Nrf2 significantly promoted the production of ROS and MDA in DOX-treated H9c2 cells, and Nrf2 knockdown reversed the protection of ORM1 in DOX-treated H9c2 cells. In conclusion, inhibition of Nrf2 activation reversed the antioxidative effects of ORM1 against DOX-induced myocardial toxicity in H9c2 cells (Figures 4(d)–4(f)). TUNEL analysis showed that Nrf2 siRNA reversed the protective effects of ORM1 in DOX-treated H9c2 cells (Figures 4(g) and 4(h)). These findings confirmed that the Nrf2 pathway was involved in the protective function of ORM1 in cardiomyocytes affected by DOX-induced oxidative stress and apoptosis.

## 4. Discussion

DOX has been used for more than half a century as an anticancer drug and is the cornerstone of chemotherapy in children and adults; however, its cardiotoxicity poses a serious threat. Accumulating evidence shows that oxidative stress and cell apoptosis play key roles in the pathogenesis of DOX-induced myocardial injury [23, 24], which is caused by the elevated production of ROS and 4-HNE (the final product of lipid peroxidation [25, 26]) and leads to cardiomyocyte apoptosis.

In our study, LC/MS technology was used to identify and quantify candidate proteins that are differentially expressed in response to DOX-induced cardiotoxicity. We found that ORM1 levels were significantly decreased, and analyses of in vivo and in vitro DOX-induced myocardial injury models confirmed this result. We also found that the levels of oxidative stress and apoptosis were increased and that ORM1 inhibited oxidative stress and apoptosis in H9C2 cells via Nrf2 signaling. Many studies have shown that various molecular mechanisms and signaling pathways can regulate oxidative stress and cardiomyocyte apoptosis; for example, activation of the Nrf2/HO-1 pathway can affect DOX-induced myocardial injury [19–21]; our study produced consistent findings and showed that the Nrf2/HO-1 pathway also participates in the antiapoptotic action of ORM1 in DOX-treated H9c2 cells.

Nrf2 is the master regulator of cellular redox homeostasis and is also involved in maintaining mitochondrial redox homeostasis by providing reduced forms of glutathione (GSH) and mitochondrial antioxidant enzymes such as GSH peroxidase, superoxide dismutase, and peroxiredoxin. Nrf2 deficiency results in impaired mitochondrial fatty acid oxidation, respiration, and adenosine triphosphate (ATP) production. Recent studies have shown that Nrf2 also affects mitochondrial function in cardiomyocyte regeneration and neural stem/progenitor cell survival [27–29]. However, the function of Nrf2 in myocardial injury caused by DOX is not completely understood. In future studies, we intend to further analyze the function of Nrf2 in the mitochondria of DOX-treated mice.

The addition of exogenous ORM1 reduced both the oxidative stress and apoptosis caused by DOX. DOX-induced oxidative stress was significantly reversed when H9c2 cells were treated with a combination of ORM1 and DOX, confirming the antioxidative power of ORM1. Cardiomyocyte apoptosis is another hallmark of acute DOX-induced cardiotoxicity, and ORM1 reportedly exhibits protective effects against apoptosis under various pathogenic conditions [12–14]. The results from the TUNEL assay confirmed the antiapoptotic effect of ORM1, as the protein significantly inhibited DOX-induced apoptosis in vitro. Nrf2 silencing negated the protective effects of ORM1 against DOX-induced cardiomyocyte injury, suggesting that ORM1 regulates DOX-induced cardiomyocyte oxidation and apoptosis by upregulating Nrf2/HO-1 signaling and other important antioxidative Nrf2-related pathways that influence cell apoptosis [30, 31].

There were several limitations to this study. First, the effects of ORM1 on DOX-induced oxidative stress and apoptosis in cardiomyocytes were only examined in vitro. ORM1 cardiac-specific transgenic mice or knockout mice will be considered in future studies to clarify the key role of ORM1 in vivo. Moreover, it is not clear whether there was an Nrf2 binding site in the promoter region of the ORM1 gene. Chromatin immunoprecipitation and luciferase reporter gene assays will be used to clarify the details of the relationship between ORM1 and Nrf2.

## 5. Conclusions

In summary, our study showed that ORM1 could attenuate DOX-induced oxidative stress and apoptosis in cardiomyocytes via Nrf2 signaling, suggesting that ORM1 might be an effective therapeutic target for the treatment of DOX-induced cardiotoxicity. This finding provides novel insights that may contribute to significant advances in the field of oncology.

## Data Availability

The data used in this study are available upon request from the corresponding author.

## Supplementary Materials

Supplementary Materials Fig. S1: DOX causes heart failure on C57BL/6 mice. (a) The level of FS valve (*n* = 20). (b) Serum LDH (*n* = 10). (c) Serum CK/MB (*n* = 10). (d) Relative ANP mRNA levels of hearts (*n* = 10). Data are expressed as the mean ± standard error of the mean (SEM); ^∗∗^*P* < 0.01. Fig S2: DOX causes a dose-dependent inhibition of cell viability in H9c2 cells (*n* = 3). Data are expressed as the mean ± standard error of the mean (SEM); ^∗^*P* < 0.05 and ^∗∗^*P* < 0.01. Fig. S3: ORM1 upregulated cell viability in DOX-induced cardiomyocytes. (a) The cell viability with different dose ORM1 (*n* = 3). (b) ORM1 upregulated cell viability in DOX-induced cardiomyocytes (*n* = 3). Data are expressed as the mean ± standard error of the mean (SEM); ^∗∗^*P* < 0.01. Fig S4: sequences of the primers used for real-time RT-PCR analysis.
