# Corrigendum to “Orosomucoid 1 Attenuates Doxorubicin-Induced Oxidative Stress and Apoptosis in Cardiomyocytes via Nrf2 Signaling”

**DOI:** 10.1155/2022/9828942

**Published:** 2022-07-04

**Authors:** Xiaoli Cheng, Dan Liu, Ruinan Xing, Haixu Song, Xiaoxiang Tian, Chenghui Yan, Yaling Han

**Affiliations:** ^1^Department of Cardiology, Shengjing Hospital of China Medical University, Shenyang, Liaoning Province 110004, China; ^2^Department of Cardiology and Cardiovascular Research Institute of PLA, General Hospital of Northern Theater Command, Shenyang, Liaoning Province 110016, China

In the article titled “Orosomucoid 1 Attenuates Doxorubicin-Induced Oxidative Stress and Apoptosis in Cardiomyocytes via Nrf2 Signaling” [1], there are errors in the cleaved caspase-3 panel of Figure 4(a) and the DOX+Nrf2 siRNA panel of Figure 4(g).

The authors mistakenly uploaded the incorrect Figure 4(g), while the error in Figure 4(a) was inadvertently introduced during the production process. The corrected Figure 4 is as follows.

## Figures and Tables

**Figure 1:**
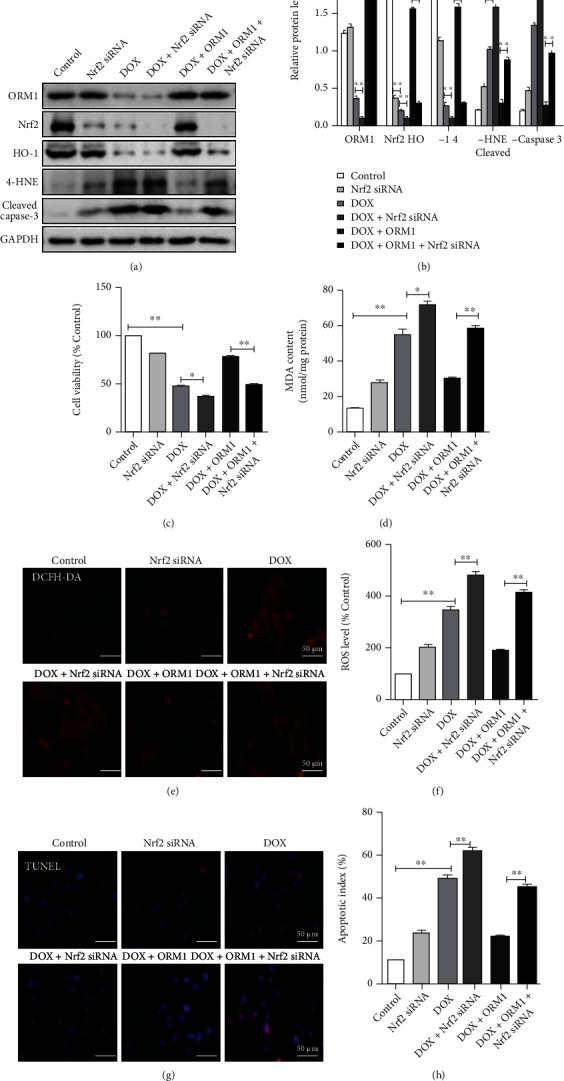
Nrf2 knockdown reverses the protective effects of ORM1 in doxorubicin- (DOX-) treated H9c2 cells. (a, b) Western blot analysis of ORM1, Nrf2, HO-1, 4-HNE, and cleaved caspase-3. (c) Cell survival analysis using the Cell Counting Kit 8 (CCK-8). (d) Cellular malondialdehyde (MDA) content. (e, f) Fluorescence image (red fluorescence) of reactive oxygen species (ROS) measured using dichlorodihydrofluorescein diacetate (DCFH-DA). (g, h) Terminal deoxynucleotidyl transferase dUTP nick end labeling (TUNEL) staining images with calculated apoptosis indices. Data are expressed as the mean ± standard error of the mean (SEM); *n* = 6. ^∗∗^*P* < 0.01 and ^∗^*P* < 0.05.
