# Supplementary material for: Orosomucoid 1 Attenuates Doxorubicin-Induced Oxidative Stress and Apoptosis in Cardiomyocytes via Nrf2 Signaling
Source: Biomed Res Int. 2020 Oct 19;2020:5923572. doi: 10.1155/2020/5923572 (PMC7591952; doi:10.1155/2020/5923572)
Supplement: Supplementary Materials — Fig. S1: DOX causes heart failure on C57BL/6 mice. (a) The level of FS valve (n = 20). (b) Serum LDH (n = 10). (c) Serum CK/MB (n = 10). (d) Relative ANP mRNA levels of hearts (n = 10). Data are expressed as the mean ± standard error of the mean (SEM); ∗∗P < 0.01. Fig S2: DOX causes a dose-dependent inhibition of cell viability in H9c2 cells (n = 3). Data are expressed as the mean ± standard error of the mean (SEM); ∗P < 0.05 and ∗∗P < 0.01. Fig. S3: ORM1 upregulated cell viability in DOX-induced cardiomyocytes. (a) The cell viability with different dose ORM1 (n = 3). (b) ORM1 upregulated cell viability in DOX-induced cardiomyocytes (n = 3). Data are expressed as the mean ± standard error of the mean (SEM); ∗∗P < 0.01. Fig S4: sequences of the primers used for real-time RT-PCR analysis. [file 5923572.f1.zip › 5923572-editable supplementary Figure 3.pdf]

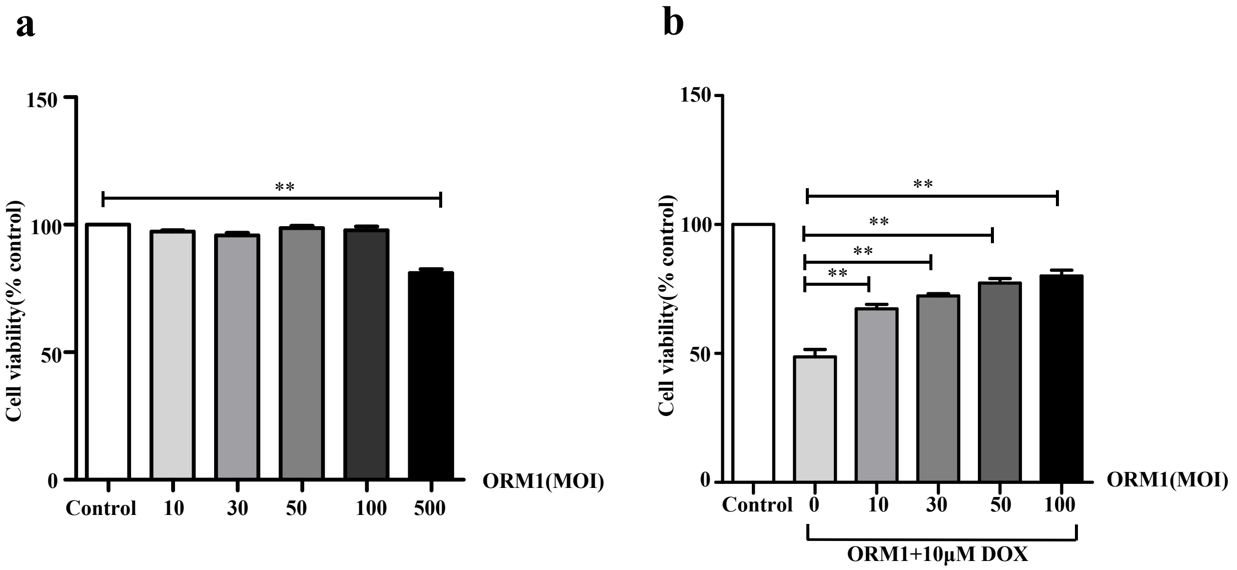

Fig. S3. ORM1 upregulated cell viability in DOX-induced cardiomyocytes
(a) The cell viability with different dose ORM1 (n=3). (b) ORM1 upregulated cell viability in DOX-induced cardiomyocytes(n=3). Data are expressed as the mean ±standard error of the mean (SEM),**P<0.01.
